# Bayesian Power‐Based Sample Size Determination for Diagnostic Accuracy Studies

**DOI:** 10.1002/pst.70122

**Published:** 2026-09-20

**Authors:** Shunsuke Shiota, Go Horiguchi, Akari Naito, Mitsuko Nakata, Satoshi Teramukai

**Affiliations:** ^1^ Department of Biostatistics, Graduate School of Medical Science Kyoto Prefectural University of Medicine Kyoto Japan

**Keywords:** analysis prior, Bayesian power, design prior, diagnostic accuracy study, sample size determination

## Abstract

In diagnostic accuracy studies, it is crucial to estimate the required sample size beforehand for the appropriate evaluation of diagnostic measures (e.g., sensitivity and/or specificity). In the development of pharmaceuticals and medical devices, although frequentist approaches have been used for sample size determination, Bayesian approaches have gained attention recently. We propose a Bayesian method for sample size determination using two kinds of priors (i.e., analysis prior and design prior) based on “Bayesian power.” In the proposed method, an analysis prior and a design prior were introduced for the sensitivity and/or specificity parameters and a design prior for the prevalence parameter. For each possible future dataset at a given sample size, the analysis prior was updated to calculate the posterior probability of satisfying a prespecified success criterion. The design priors were used to obtain the prior predictive distribution of the future data, and Bayesian power was calculated as the prior predictive probability of satisfying the success criterion. Sample size determination was performed by evaluating Bayesian power over a range of candidate sample sizes according to a prespecified criterion. Numerical results showed that, under the parameter settings considered, the required sample size generally decreased as uncertainty in the design priors decreased when their means were above the corresponding target values. With the effective sample size of the analysis prior fixed, a more optimistic analysis prior reduced the required sample size. We also evaluated the method for joint sensitivity and specificity criteria and performed comparisons with commonly used frequentist methods. These results demonstrate how uncertainty represented by the design priors and information represented by the analysis prior affect Bayesian power and the required sample size.

## 1 Introduction

Diagnostic accuracy studies evaluate whether a diagnostic trial can correctly identify the presence or absence of a disease in a subject. Diagnostic accuracy can be evaluated using various measures, including sensitivity, specificity, positive and negative predictive values, likelihood ratios, and the area under the receiver operating characteristic (ROC) curve. It is crucial to determine the sample size beforehand to estimate these measures with appropriate accuracy while ensuring the ethical conduct, efficiency, and scientific validity of the study. In general, the sample size of a clinical study should not be too small or too large. If the sample size is too small, the precise estimation of treatment effect is hindered by random variation. By contrast, if the sample size is too large, ethical issues such as exposing a large number of subjects to an invasive diagnostic trial or financial issues may arise.

Frequentist methods have been used extensively in drug and device development. Standard frequentist approaches in sample size determination for diagnostic accuracy studies are based on hypothesis testing or confidence interval length. In the one‐sample design with hypothesis testing, let the sensitivity parameters under the null hypothesis and the alternative hypothesis be $\theta_{0}$ and $\theta_{1}$, respectively. When π is the prevalence of the disease, the total sample size n with one‐sided significance level α and power 1−β can be obtained by the following equation:

(1) $$n=\frac{{\left[z_{1-\alpha }\sqrt{\theta_{0}\left(1-\theta_{0}\right)}+z_{1-\beta }\sqrt{\theta_{1}\left(1-\theta_{1}\right)}\right]}^{2}}{\pi {\left(\theta_{1}-\theta_{0}\right)}^{2}}.$$

where $z_{1-\alpha }$ and $z_{1-\beta }$ are 1−α and 1−β quantiles of the standard normal distribution, respectively [1]. These calculations condition on fixed planning values for diagnostic accuracy and prevalence. In practice, however, those quantities are uncertain at the design stage, particularly when evidence comes from small pilot studies, related populations, or expert judgments.

Meanwhile, Bayesian methods in clinical studies have gained attention recently. In fact, many Bayesian methods have already been applied to the regulatory decision for diagnostic devices in the United States [2]. Bayesian sample size determination can propagate the pre‐experimental uncertainty through the prior predictive distribution. Wilson et al. [3] proposed the Bayesian approach to sample size determination for diagnostic accuracy studies in which the priors for sensitivity and prevalence are assumed, and the sample size is determined based on the length of the credible interval of the posterior distribution of the sensitivity parameter. A Bayesian approach has also been applied to planning diagnostic accuracy studies for point‐of‐care COVID‐19 tests, illustrating how external laboratory information can be propagated into study design [4]. In this article, we refer to the unconditional prior predictive probability that a future study satisfies a prespecified Bayesian success criterion as “Bayesian power” [5, 6, 7].

In this article, we propose an innovative Bayesian power‐based sample size determination method incorporating two distinct priors: the analysis prior and the design prior [8, 9, 10]. This method adopts the idea of calculating the prior predictive probability of a “significant” trial result using two different priors. The analysis prior represents the distribution assumed for posterior inference and decision‐making, whereas the design prior represents uncertainty about plausible parameter values at the planning stage and is used to evaluate the prior predictive probability of achieving that success criterion.

In Bayesian diagnostic accuracy studies, the analysis prior and the design prior are used for the sensitivity parameter, and another design prior is used for the prevalence parameter. Using these priors, the posterior probability and the prior predictive distribution are obtained, and the required sample size is determined based on the Bayesian power [7]. The proposed method is implemented in an R Shiny application (see Appendix A).

This article is organized as follows. Section 2 describes the proposed method in detail, and Section 3 evaluates its properties under various settings. In Section 4, we compare the sample sizes obtained using standard frequentist methods with those obtained under a limiting case of the proposed method, in which degenerate design priors are used. Section 5 extends the proposed method to accommodate specificity. Section 6 presents an application example, and Section 7 discusses the findings.

## 2 Methods

### 2.1 Notation

Suppose that a study includes n subjects in total. Let $n_{d}$ and $n_{k}$ be the numbers of subjects with and without the disease, respectively, so that $n=n_{d}+n_{k}$. Among the $n_{d}$ subjects with the disease, let $n_{s}$ be the number of true‐positive cases. Among the $n_{k}$ subjects without the disease, let $n_{c}$ be the number of true‐negative cases. Sensitivity and specificity are defined as θ=Pr(test postive|disease present) and η=Pr(test negative|disease absent), respectively, and are commonly estimated by $n_{s}/n_{d}$ and $n_{c}/n_{k}$. Prevalence is defined as ρ=Pr(disease present) and is commonly estimated by $n_{d}/n$.

### 2.2 Bayesian Power‐Based Sample Size Determination

Let $f_{n_{d}}\left(n_{s}|\theta \right)$ denote the probability mass function of $n_{s}$ conditional on $n_{d}$ and θ, where $n_{s}\;|\;n_{d},\theta ∼\mathrm{Bin}\left(n_{d},\theta \right)$. Let $\pi^{A}\left(\theta \right)$ denote the density of the analysis prior $\theta ∼\text{Beta}\left(a_{\theta }^{A},b_{\theta }^{A}\right)$. In Bayesian conjugate analysis, after observing $n_{d}$ and $n_{s}$, the analysis prior is updated to the posterior $\theta \;|\;n_{d},n_{s}∼\text{Beta}\left(a_{\theta }^{A}+n_{s},b_{\theta }^{A}+n_{d}-n_{s}\right)$, and the posterior probability that the sensitivity parameter exceeds the prespecified target value $\theta^{*}$ is obtained by

(2) $$\mathrm{Pr}\left(\theta >\theta^{*}|n_{d},n_{s}\right)=\int_{\theta^{*}}^{1}\pi^{A}\left(\theta |n_{d},n_{s}\right)d\theta .$$

Let $\pi^{D}\left(\theta \right)$ denote the density of the design prior $\theta ∼\text{Beta}\left(a_{\theta }^{D},b_{\theta }^{D}\right)$. The prior predictive distribution of $n_{s}$ follows a beta‐binomial distribution expressed as $\int_{0}^{1}f_{n_{d}}\left(n_{s}|\theta \right)\pi^{D}\left(\theta \right)d\theta$. Let $\lambda_{\theta }$ be the prespecified probability threshold for sensitivity and A be the set of $n_{s}$ satisfying $\mathrm{Pr}\left(\theta >\theta^{*}|n_{d},n_{s}\right)>\lambda_{\theta }$. The prior predictive probability that $n_{s}$ is observed in A is $\beta_{\theta }\left(n_{d}\right)=\sum_{n_{s}\in A}\int_{0}^{1}f_{n_{d}}\left(n_{s}|\theta \right)\pi^{D}\left(\theta \right)d\theta$, where $\beta_{\theta }\left(\cdot \right)$ is a Bayesian power function considering only sensitivity.

Then, we describe the proposed Bayesian power‐based method for sample size determination incorporating the prevalence parameter. Let $f_{n}\left(n_{d}|\rho \right)$ denote the probability mass function of $n_{d}$ conditional on n and ρ, where $n_{d}\;|\;n,\rho ∼\mathrm{Bin}\left(n,\rho \right)$. Let $\pi^{D}\left(\rho \right)$ denote the density of the design prior $\rho ∼\text{Beta}\left(a_{\rho }^{D},b_{\rho }^{D}\right)$. The prior predictive distribution of $n_{d}$ follows a beta‐binomial distribution expressed as $\int_{0}^{1}f_{n}\left(n_{d}|\rho \right)\pi^{D}\left(\rho \right)d\rho$. As described above, for each value of $n_{d}$, the prior predictive probability mass function of $n_{s}$ is $\int_{0}^{1}f_{n_{d}}\left(n_{s}|\theta \right)\pi^{D}\left(\theta \right)d\theta$. Therefore, under a fixed total sample size n, the prior predictive probability for $n_{d}$ and $n_{s}$ is given by

(3) $$\mathrm{pp}\left(n_{d},n_{s}\right)=\int_{0}^{1}f_{n}\left(n_{d}|\rho \right)\pi^{D}\left(\rho \right)d\rho \cdot \int_{0}^{1}f_{n_{d}}\left(n_{s}|\theta \right)\pi^{D}\left(\theta \right)d\theta .$$

Equation (2) can be used to calculate the posterior probability $\mathrm{Pr}\left(\theta >\theta^{*}|n_{d},n_{s}\right)$ for a set of observable data $\left(n_{d},n_{s}\right)$ with a total sample size of n. For a prespecified probability threshold for sensitivity $\lambda_{\theta }$, we declare the study successful with respect to sensitivity if $\mathrm{Pr}\left(\theta >\theta^{*}|n_{d},n_{s}\right)>\lambda_{\theta }$. Let B be the set of $\left(n_{d},n_{s}\right)$ satisfying $\mathrm{Pr}\left(\theta >\theta^{*}|n_{d},n_{s}\right)>\lambda_{\theta }$. Using Equation (3), the prior predictive probability that $\left(n_{d},n_{s}\right)$ is observed in B is given by

(4) $$\beta_{\theta ,\rho }\left(n\right)=\sum_{\left(n_{d},n_{s}\right)\in B}\mathrm{pp}\left(n_{d},n_{s}\right).$$

We refer to $\beta_{\theta ,\rho }\left(n\right)$ as the Bayesian power function [7]. In contrast to conventional frequentist power, which is evaluated conditional on fixed parameter values, the Bayesian power defined here is the prior predictive probability of meeting the prespecified posterior‐probability success criterion, averaged over the design priors. $\beta_{\theta ,\rho }\left(n\right)$ has a saw‐tooth shape and is not monotonically increasing since the prior predictive probability is derived based on the discrete probability function [9]. For a probability threshold γ, the smallest value is selected as the required sample size N for a study such that $\beta_{\theta ,\rho }\left(n\right)\geq \gamma ,\forall n\geq N$.

In summary, the method for calculating Bayesian power under a fixed n is as follows:

1. Enumerate all possible pairs of $\left(n_{d},n_{s}\right)$ (there are (n+2)(n+1)/2 combinations).
2. For each $\left(n_{d},n_{s}\right)$, calculate the posterior probability of exceeding $\theta^{*}$ using Equation (2).
3. Define the set B as the collection of $\left(n_{d},n_{s}\right)$ satisfying $\mathrm{Pr}\left(\theta >\theta^{*}|n_{d},n_{s}\right)>\lambda_{\theta }$.
4. Determine the probability that $\left(n_{d},n_{s}\right)$ is observed in B using Equations (3) and (4).

## 3 Numerical Results

We examined how the sample size changed as these parameters were varied. Three analysis priors were prepared with an effective sample size of 2. We assumed beta distributions with means of 0.5, 0.7, and 0.9 as follows: $\pi^{A}\left(\theta \right)=\text{Beta}\left(1,1\right)$, Beta(1.4,0.6), and Beta(1.8,0.2), respectively. In this study, unless otherwise noted, the posterior probability threshold $\lambda_{\theta }=0.9$ and the Bayesian power threshold γ=0.8 were used. It is practical to determine the design prior based on the sponsor's expectations of diagnostic trial performance and elicitation of expert opinion. To translate such information into the design priors for prevalence and sensitivity, we considered two convenient parameterizations commonly used in prior elicitation: specification through the mean and the effective sample size, and specification through probability intervals. These two specifications correspond to established classes of elicitation methods reviewed by Garthwaite et al. [11].

### 3.1 Specification of the Design Prior Using the Mean and the Effective Sample Size

The beta distribution Beta(a,b) has mean a/(a+b) and variance $\mathrm{ab}/{\left(a+b\right)}^{2}\left(a+b+1\right)$. If a=mp and b=m(1−p), then the beta distribution has mean p and variance p(1−p)/(m+1). Thus, m can be interpreted as a parameter that controls the variance, with larger values corresponding to smaller variance. Furthermore, since a+b=m, m also represents the effective sample size. In this section, the parameters of the design prior were transformed into variables $a_{\rho }^{D}=m_{\rho }p_{\rho },\;b_{\rho }^{D}=m_{\rho }\left(1-p_{\rho }\right),\;a_{\theta }^{D}=m_{\theta }p_{\theta },\;b_{\theta }^{D}=m_{\theta }\left(1-p_{\theta }\right)$, and the sample size was calculated by varying the values of $m_{\rho },p_{\rho },m_{\theta },p_{\theta }$. The results of numerical calculations are shown in Tables 1, 2, 3. Under the parameter settings examined, the required sample size generally decreased as the effective sample size of the design prior $\pi^{D}\left(\theta \right)$ or $\pi^{D}\left(\rho \right)$ increased. As the mean of the analysis prior $\pi^{A}\left(\theta \right)$ increased, the sample size also decreased.

**TABLE 1:** Required sample sizes for several specifications of the design priors $\pi^{D}\left(\theta \right)$ and $\pi^{D}\left(\rho \right)$, assuming the analysis prior $\pi^{A}\left(\theta \right)=\text{Beta}\left(1,1\right)$.

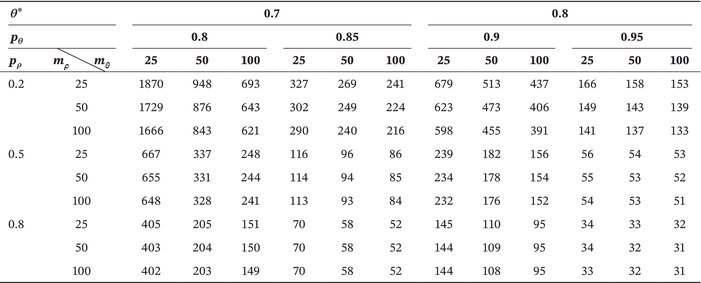

*Note:* $\theta^{*}$ is the target value for sensitivity. $p_{\theta }$ and $p_{\rho }$ are the means of the design priors $\pi^{D}\left(\theta \right)$ and $\pi^{D}\left(\rho \right)$, respectively. $m_{\theta }$ and $m_{\rho }$ are the effective sample sizes of the design priors $\pi^{D}\left(\theta \right)$ and $\pi^{D}\left(\rho \right)$, respectively.

**TABLE 2:** Required sample sizes for several specifications of the design priors $\pi^{D}\left(\theta \right)$ and $\pi^{D}\left(\rho \right)$, assuming the analysis prior $\pi^{A}\left(\theta \right)=\text{Beta}\left(1.4,0.6\right)$.

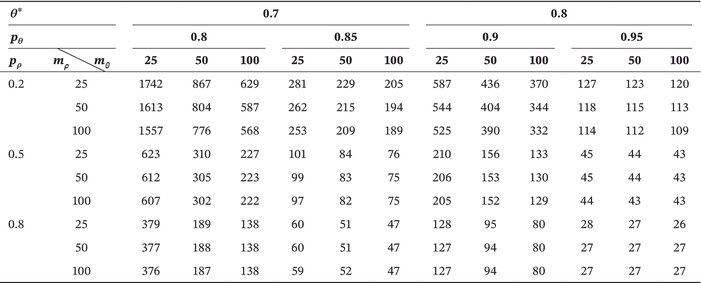

*Note:* $\theta^{*}$ is the target value for sensitivity. $p_{\theta }$ and $p_{\rho }$ are the means of the design priors $\pi^{D}\left(\theta \right)$ and $\pi^{D}\left(\rho \right)$, respectively. $m_{\theta }$ and $m_{\rho }$ are the effective sample sizes of the design priors $\pi^{D}\left(\theta \right)$ and $\pi^{D}\left(\rho \right)$, respectively.

**TABLE 3:** Required sample sizes for several specifications of the design priors $\pi^{D}\left(\theta \right)$ and $\pi^{D}\left(\rho \right)$, assuming the analysis prior $\pi^{A}\left(\theta \right)=\text{Beta}\left(1.8,0.2\right)$.

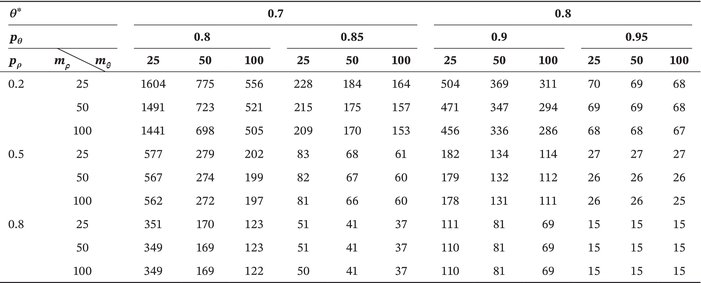

*Note:* $\theta^{*}$ is the target value for sensitivity. $p_{\theta }$ and $p_{\rho }$ are the means of the design priors $\pi^{D}\left(\theta \right)$ and $\pi^{D}\left(\rho \right)$, respectively. $m_{\theta }$ and $m_{\rho }$ are the effective sample sizes of the design priors $\pi^{D}\left(\theta \right)$ and $\pi^{D}\left(\rho \right)$, respectively.

**FIGURE 1:**
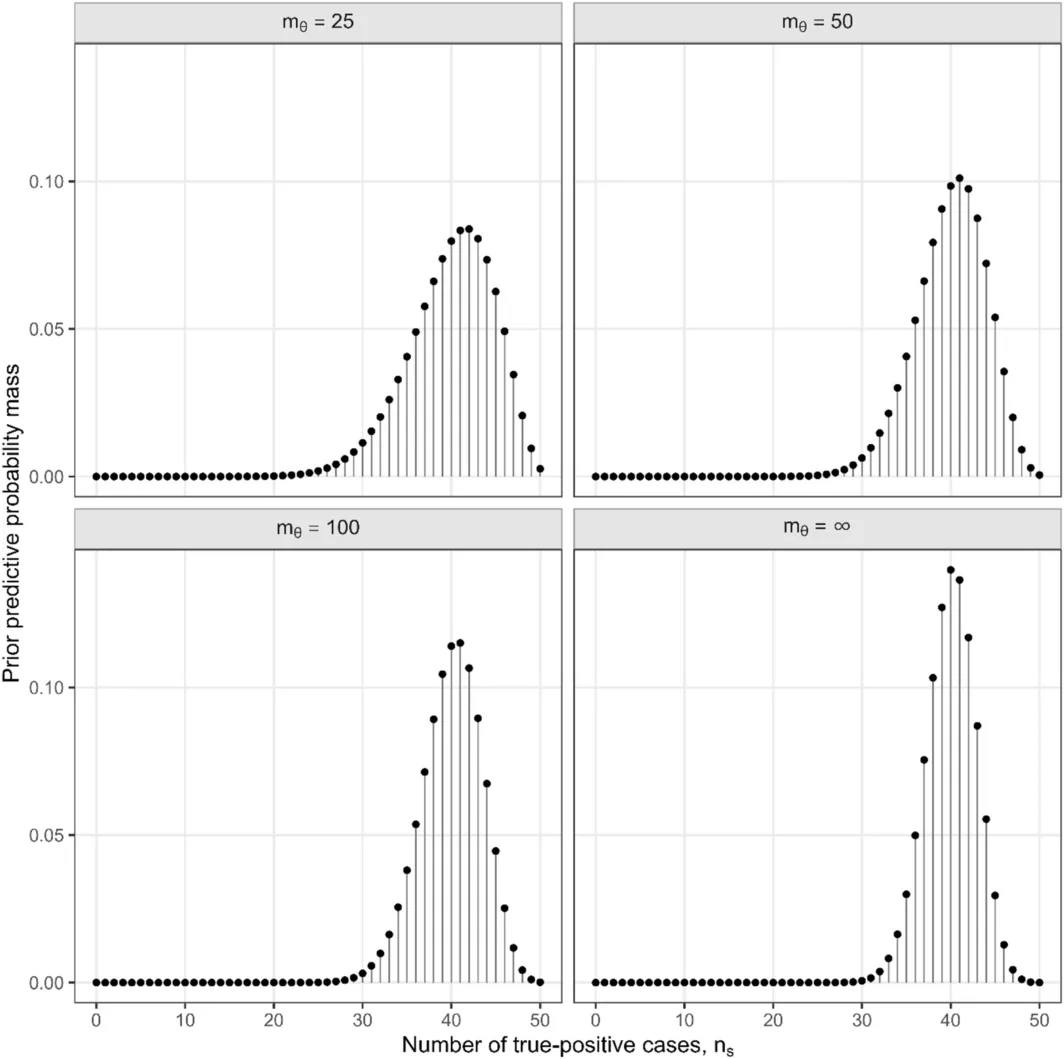
Prior predictive distributions of the number of true‐positive cases $n_{s}$ for several values of the effective sample size of the design prior $\pi^{D}\left(\theta \right)$ $(m_{\theta }=25,50,100$, and ∞). The mean of the design prior $\pi^{D}\left(\theta \right)$ is fixed at $p_{\theta }=0.8$, and the number of subjects with the disease $n_{d}$ is fixed at 50.

To illustrate the role of the effective sample size $m_{\theta }$, Figure 1 shows the prior predictive distributions of the number of true‐positive cases $n_{s}$ for several values of $m_{\theta }$. In this example, the mean of the design prior for sensitivity was fixed at $p_{\theta }=0.8$ and the number of subjects with the disease $n_{d}$ was fixed at 50. For finite values of $m_{\theta }$, the prior predictive distribution follows a beta‐binomial distribution. Smaller values of $m_{\theta }$ produce more dispersed prior predictive distributions, reflecting greater uncertainty about the anticipated sensitivity at the design stage. In contrast, as $m_{\theta }$ increases, the prior predictive distribution becomes increasingly concentrated around $n_{d}p_{\theta }$. In the limiting case where $m_{\theta }=\infty$, the design prior degenerates to $p_{\theta }$ (i.e., a degenerate distribution), and the prior predictive distribution reduces to the binomial distribution $\mathrm{Bin}\left(n_{d},p_{\theta }\right)$. These results illustrate that $m_{\theta }$ controls the degree of uncertainty incorporated into the design prior and, consequently, into the Bayesian power calculation.

**FIGURE 2:**
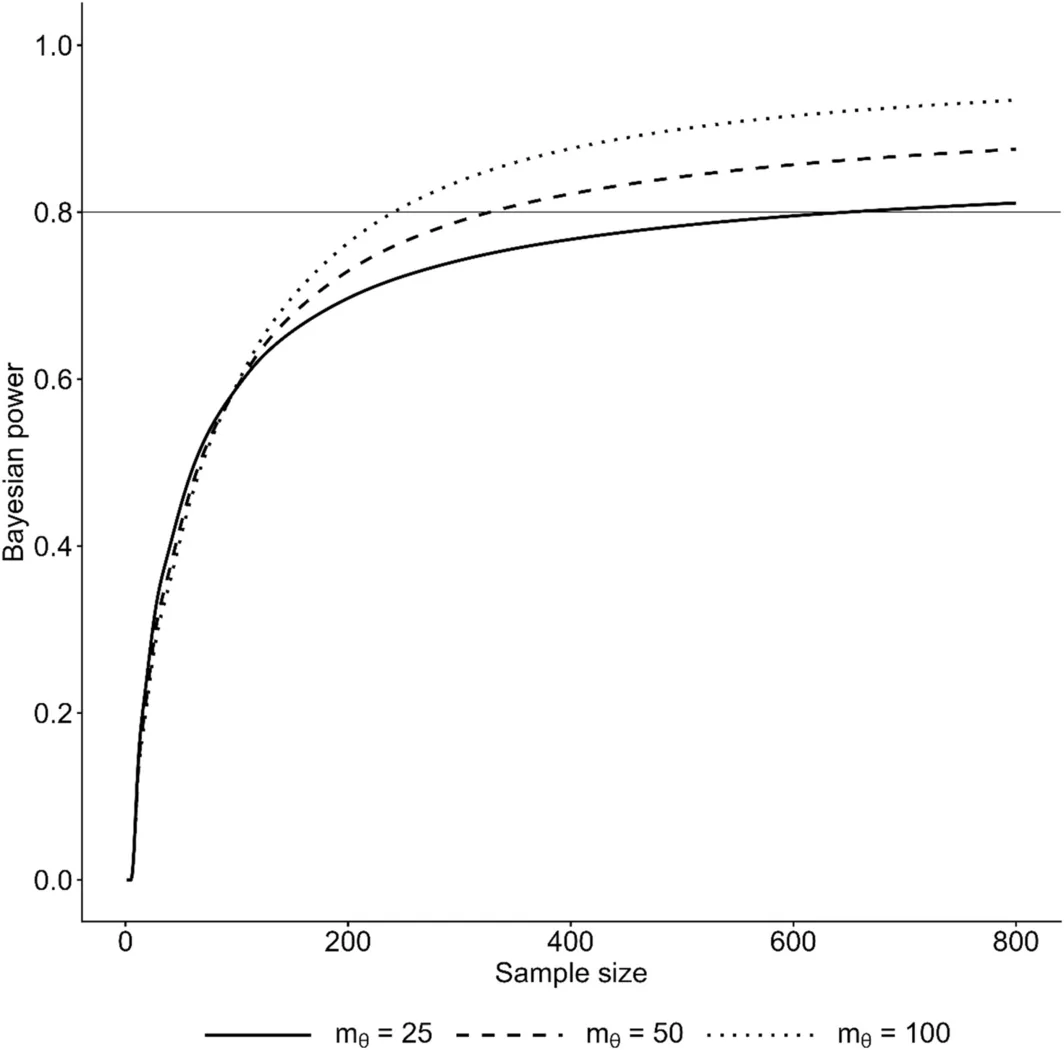
Bayesian power functions $\beta_{\theta ,\rho }\left(n\right)$ for several values of the effective sample size of the design prior $\pi^{D}\left(\theta \right)$ $(m_{\theta }=25,50,$ and 100). Other parameter values are $\theta^{*}=0.7,\pi^{A}\left(\theta \right)=\text{Beta}\left(1,1\right),\;p_{\theta }=0.8,m_{\rho }=100$ and $p_{\rho }=0.5$.

This uncertainty in the design prior is propagated to the Bayesian power calculation. Figure 2 illustrates the power function when $\theta^{*}=0.7,\pi^{A}\left(\theta \right)=\text{Beta}\left(1,1\right),p_{\theta }=0.8,\;m_{\rho }=100,\;p_{\rho }=0.5,$ and $m_{\theta }=25,50$, and 100, respectively. As the value of $m_{\theta }$ decreases, the power function also exhibits a more gradual increase. In fact, following the argument of Eaton et al. [12], the Bayesian power converges to the design‐prior probability that the sensitivity exceeds the target value, that is, $\int_{\theta^{*}}^{1}\pi^{D}\left(\theta \right)d\theta$. These results indicate that, under the parameter settings considered here, greater uncertainty in the design prior for sensitivity leads to a more gradual increase in Bayesian power and may require a larger sample size.

### 3.2 Specification of the Design Prior Using Equal‐Tail Interval

If prevalence and sensitivity are assumed to lie within $l_{\rho }\leq \rho \leq u_{\rho }$ and $l_{\theta }\leq \theta \leq u_{\theta }$, respectively, the design priors can be determined such that the intervals $L_{\rho }=\left[l_{\rho },u_{\rho }\right]$ and $L_{\theta }=\left[l_{\theta },u_{\theta }\right]$ are equal‐tail intervals of $1-\alpha_{\rho }$ and $1-\alpha_{\theta }$ levels, respectively. In other words, obtain $\left(a_{\rho }^{D},b_{\rho }^{D}\right)$ and $\left(a_{\theta }^{D},b_{\theta }^{D}\right)$ that satisfy the following equations [13].

(5) $$\int_{0}^{l_{\rho }}\pi^{D}\left(\rho \right)d\rho =\int_{u_{\rho }}^{1}\pi^{D}\left(\rho \right)d\rho =\frac{\alpha_{\rho }}{2},\;\int_{0}^{l_{\theta }}\pi^{D}\left(\theta \right)d\theta =\int_{u_{\theta }}^{1}\pi^{D}\left(\theta \right)d\theta =\frac{\alpha_{\theta }}{2}.$$

The numerical solution of Equation (5) can be obtained by using the beta.select function included in the LearnBayes package of R [14]. We set $\alpha =\alpha_{\rho }=\alpha_{\theta }=0.05$ and calculated the sample size by varying the values of $l_{\rho },u_{\rho },l_{\theta },$ and $u_{\theta }$. The results are shown in Table 4.

**TABLE 4:** Required sample sizes for several equal‐tail interval specifications of the design priors $\pi^{D}\left(\theta \right)$ and $\pi^{D}\left(\rho \right)$.

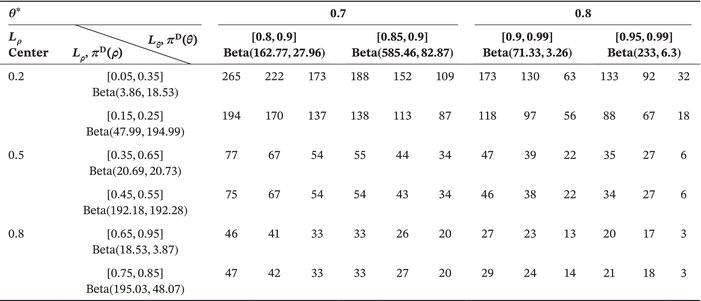

*Note:* $\theta^{*}$ is the target value for sensitivity. From left to right are the sample sizes when the analysis priors $\pi^{A}\left(\theta \right)$ are Beta(1,1), Beta(1.4,0.6) and Beta(1.8,0.2), respectively. $L_{\theta }$ and $L_{\rho }$ are the 95% equal‐tail credible intervals of the design priors $\pi^{D}\left(\theta \right)$ and $\pi^{D}\left(\rho \right)$, respectively.

## 4 Comparison With Frequentist Sample Size Methods

To evaluate the properties of the proposed method, we compared the sample sizes obtained using the proposed method with those calculated using three frequentist approaches: Approach 1 is a normal approximation method to obtain the total sample size n using Equation (1); Approach 2 and Approach 3 are Method 0 and Method 3 in Li and Fine [15], respectively. Let α be the one‐sided significance level, 1−β be the statistical power, π be the prevalence of the disease, and $\theta_{0}$ and $\theta_{1}$ be the null hypothesis and the alternative hypothesis for sensitivity, respectively.

Approach 2 is similar to Approach 1, but uses an exact probability based on a binomial distribution rather than a normal approximation. The smallest $n_{d}$ that satisfies the following inequalities is selected:

(6) $$\left\{\begin{array}{l} \sum_{n_{s}=c^{F}\left(n_{d}\right)}^{n_{d}}f_{n_{d}}\left(n_{s}|\theta_{0}\right)\leq \alpha \\ \sum_{n_{s}=c^{F}\left(n_{d}\right)}^{n_{d}}f_{n_{d}}\left(n_{s}|\theta_{1}\right)\geq 1-\beta \end{array}\right..$$

where $c^{F}\left(n_{d}\right)$ is a critical value. Since $n_{d}$ and $n_{s}$ are discrete values, a practical way to find $n_{d}$ is to find $c^{F}\left(n_{d}\right)$ that maximizes the actual level to the extent that the nominal level α is not exceeded, and then use $c^{F}\left(n_{d}\right)$ to calculate whether the power exceeds 1−β with $n_{d}$ fixed. This naïve approach to finding the sample size is to take $n=n_{d}/\pi$.

In the comparison considered here, Approach 3 is structurally comparable to the proposed method under degenerate design priors because both use the joint distribution of $n_{d}$ and $n_{s}$ to determine the critical region and sample size. The smallest n that satisfies the following inequalities is selected:

(7) $$\left\{\begin{array}{l} \sum_{n_{d}=0}^{n}\sum_{n_{s}=c^{F}\left(n_{d}\right)}^{n_{d}}f_{n}\left(n_{d}|\rho \right)\cdot f_{n_{d}}\left(n_{s}|\theta_{0}\right)\leq \alpha \\ \sum_{n_{d}=0}^{n}\sum_{n_{s}=c^{F}\left(n_{d}\right)}^{n_{d}}f_{n}\left(n_{d}|\rho \right)\cdot f_{n_{d}}\left(n_{s}|\theta_{1}\right)\geq 1-\beta \end{array}\right..$$

where $c^{F}\left(n_{d}\right)$ is a critical value which is not common for all $n_{d}$, but depends on each $n_{d}$. That is, the idea is to find $c^{F}\left(n_{d}\right)$ for each $n_{d}$ so as to maximize the power of the whole trial. This idea reflects the practice of selecting a critical value after observing $n_{d}$ [15]. As in Approach 2, it is realistic to find $c^{F}\left(n_{d}\right)$ and calculate the power with n fixed.

For comparison, the design priors $\pi^{D}\left(\theta \right)$ and $\pi^{D}\left(\rho \right)$ were assumed to follow a degenerate distribution ($m_{\theta }=\infty$ and $m_{\rho }=\infty$ in Section 3.1). For the sensitivity parameter, the target value $\theta^{*}$ was set as the null value ($\theta_{0}$), and the degenerate design distribution at $p_{\theta }$ was set as the alternative value ($\theta_{1}$). In addition, the analysis prior $\pi^{A}\left(\theta \right)=\text{Beta}\left(1,1\right)$, the posterior probability threshold $\lambda_{\theta }=1-\alpha =0.95$ and the power γ=1−β=0.8 were used. The degenerate design prior at $p_{\rho }$ was used for the prevalence parameter. The results of sample size calculations for the four methods including the proposed method are shown in Table 5. In all settings, the sample sizes followed a decreasing pattern, with Approach 1 giving the smallest sample size, followed by the proposed method, Approach 3, and Approach 2 giving progressively larger sample sizes. The smaller sample sizes obtained using the proposed method can be understood, under the parameter settings considered, from the fact that the one‐sided exact binomial decision rule underlying Approach 3 is mathematically equivalent to the posterior‐probability criterion based on the formal limiting improper analysis prior $\pi^{A}\left(\theta \right)=\text{Beta}\left(0,1\right)$, whereas the proposed method in Table 5 uses the analysis prior $\pi^{A}\left(\theta \right)=\text{Beta}\left(1,1\right)$. See Appendix B for a formal derivation.

**TABLE 5:** Comparison of required sample sizes obtained using the proposed method and frequentist methods.

$\theta^{*}$ or $\theta_{0}$	$p_{\theta }$ or $\theta_{1}$	$p_{\rho }$ or π	Proposed method	Frequentist Approach 1	Frequentist Approach 2	Frequentist Approach 3
0.7	0.8	0.2	607	595	650	633
		0.5	243	238	260	253
		0.8	152	149	163	158
	0.85	0.2	253	248	285	269
		0.5	101	99	114	107
		0.8	63	62	72	67
0.8	0.9	0.2	433	415	470	449
		0.5	173	166	188	180
		0.8	108	104	118	112
	0.95	0.2	172	158	185	181
		0.5	68	63	74	72
		0.8	43	40	47	45

*Note:* For the proposed method, $\theta^{*}$ is the target value for sensitivity. $p_{\theta }$ and $p_{\rho }$ are the means of the design priors $\pi^{D}\left(\theta \right)$ and $\pi^{D}\left(\rho \right)$, respectively. The analysis prior $\pi^{A}\left(\theta \right)$ is Beta(1,1). For the frequentist approaches, $\theta_{0}$ and $\theta_{1}$ are the sensitivity parameters under the null and alternative hypotheses, respectively, and π is the disease prevalence. In Table 5, $m_{\theta }=m_{\rho }=\infty$; thus, the design priors $\pi^{D}\left(\theta \right)$ and $\pi^{D}\left(\rho \right)$ are degenerate at $p_{\theta }$ and $p_{\rho }$, respectively. In each scenario, $\theta^{*}=\theta_{0}$, $p_{\theta }=\theta_{1}$, and $p_{\rho }=\pi$.

## 5 Extension to Specificity and Joint Sensitivity‐Specificity Criteria

When only specificity is considered, the Bayesian power‐based sample size determination can be formulated in the same manner as that for sensitivity described in Section 2. Let $f_{n_{k}}\left(n_{c}|\eta \right)$ denote the probability mass function of $n_{c}$ conditional on $n_{k}$ and η, where $n_{c}\;|\;n_{k},\eta ∼\mathrm{Bin}\left(n_{k},\eta \right)$. Let $\pi^{A}\left(\eta \right)$ and $\pi^{D}\left(\eta \right)$ denote the analysis prior $\eta ∼\text{Beta}\left(a_{\eta }^{A},b_{\eta }^{A}\right)$ and the design prior $\eta ∼\text{Beta}\left(a_{\eta }^{D},b_{\eta }^{D}\right)$, respectively. Then, the Bayesian power and the required sample size for specificity alone are obtained by replacing θ, $n_{d}$, $n_{s}$, $\theta^{*}$, and $\lambda_{\theta }$ in Section 2 with η, $n_{k}$, $n_{c}$, $\eta^{*}$, and $\lambda_{\eta }$, respectively.

Next, we consider studies that verify that the sensitivity and specificity simultaneously exceed both targets. For the sake of simplicity, it is assumed that the number of true positives $n_{s}$ and the number of true negatives $n_{c}$ are independent of each other. With a fixed sample size n, the prior predictive probability that the number of subjects with the disease is $n_{d}$, the number of true‐positive cases is $n_{s}$, and the number of true‐negative cases is $n_{c}$ is given by

(8) $$\begin{array}{ll} \mathrm{pp}\left(n_{d},n_{s},n_{c}\right) & =\int_{0}^{1}f_{n}\left(n_{d}|\rho \right)\pi^{D}\left(\rho \right)d\rho \cdot \int_{0}^{1}f_{n_{d}}\left(n_{s}|\theta \right)\pi^{D}\left(\theta \right)d\theta \\ & \;\cdot \int_{0}^{1}f_{n_{k}}\left(n_{c}|\eta \right)\pi^{D}\left(\eta \right)d\eta . \end{array}$$

Then we calculate the posterior probabilities, $\mathrm{Pr}\left(\theta >\theta^{*}|n_{d},n_{s}\right)$ and $\mathrm{Pr}\left(\eta >\eta^{*}|n_{k},n_{c}\right)$, for a set of observable data $\left(n_{d},n_{s},n_{c}\right)$ with a sample size of n. Let D be the set of $\left(n_{d},n_{s},n_{c}\right)$ satisfying both $\mathrm{Pr}\left(\theta >\theta^{*}|n_{d},n_{s}\right)>\lambda_{\theta }$ and $\mathrm{Pr}\left(\eta >\eta^{*}|n_{k},n_{c}\right)>\lambda_{\eta }$. Using Equation (8), the prior predictive probability that $\left(n_{d},n_{s},n_{c}\right)$ is observed in D is given by

(9) $$\beta_{\theta ,\eta ,\rho }\left(n\right)=\sum_{\left(n_{d},n_{s},n_{c}\right)\in D}\mathrm{pp}\left(n_{d},n_{s},n_{c}\right).$$

Equation (9) is a Bayesian power function that takes into account three parameters (sensitivity, specificity, and prevalence) and is used to calculate the sample size. Tables 6, 7, 8 show the numerical results for three analysis‐prior settings: Beta(1,1), Beta(1.4,0.6), and Beta(1.8,0.2). The posterior probability thresholds were $\lambda_{\theta }=0.9$, $\lambda_{\eta }=0.9$, and γ=0.8. Under the parameter settings examined, the required sample size generally decreased as the effective sample size of the design prior $\pi^{D}\left(\theta \right)$ or $\pi^{D}\left(\eta \right)$ increased. As the mean of the analysis prior $\pi^{A}\left(\theta \right)$ or $\pi^{A}\left(\eta \right)$ increased, the sample size also decreased.

**TABLE 6:** Required sample sizes for several specifications of the design priors $\pi^{D}\left(\theta \right)$ and $\pi^{D}\left(\eta \right)$, assuming the analysis priors $\pi^{A}\left(\theta \right)=\text{Beta}\left(1,1\right)$ and $\pi^{A}\left(\eta \right)=\text{Beta}\left(1,1\right)$.

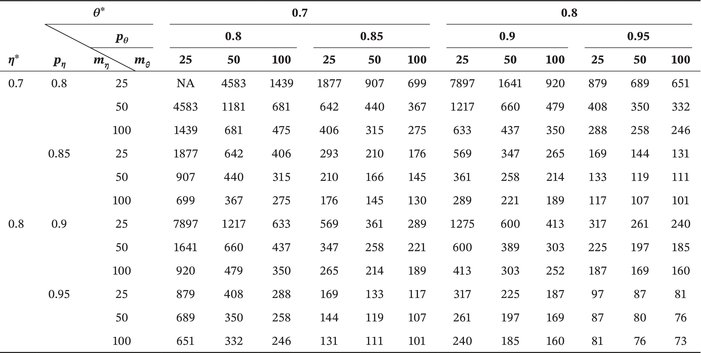

*Note:* $\theta^{*}$ and $\eta^{*}$ are the target values for sensitivity and specificity, respectively. $p_{\theta }$ and $p_{\eta }$ are the means of the design priors $\pi^{D}\left(\theta \right)$ and $\pi^{D}\left(\eta \right)$, respectively. $m_{\theta }$ and $m_{\eta }$ are the effective sample sizes of the design priors $\pi^{D}\left(\theta \right)$ and $\pi^{D}\left(\eta \right)$, respectively. The design prior $\pi^{D}\left(\rho \right)$ is fixed at $p_{\rho }=0.5$ and $m_{\rho }=100$. NA indicates that the target Bayesian power γ=0.8 is unattainable under the specified design priors $\pi^{D}\left(\theta \right)$ and $\pi^{D}\left(\eta \right)$.

**TABLE 7:** Required sample sizes for several specifications of the design priors $\pi^{D}\left(\theta \right)$ and $\pi^{D}\left(\eta \right)$, assuming the analysis priors $\pi^{A}\left(\theta \right)=\text{Beta}\left(1.4,0.6\right)$ and $\pi^{A}\left(\eta \right)=\text{Beta}\left(1.4,0.6\right)$.

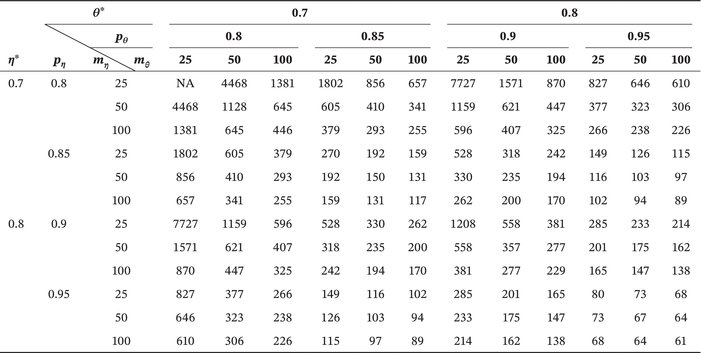

*Note:* $\theta^{*}$ and $\eta^{*}$ are the target values for sensitivity and specificity, respectively. $p_{\theta }$ and $p_{\eta }$ are the means of the design priors $\pi^{D}\left(\theta \right)$ and $\pi^{D}\left(\eta \right)$, respectively. $m_{\theta }$ and $m_{\eta }$ are the effective sample sizes of the design priors $\pi^{D}\left(\theta \right)$ and $\pi^{D}\left(\eta \right)$, respectively. The design prior $\pi^{D}\left(\rho \right)$ is fixed at $p_{\rho }=0.5$ and $m_{\rho }=100$. NA indicates that the target Bayesian power γ=0.8 is unattainable under the specified design priors $\pi^{D}\left(\theta \right)$ and $\pi^{D}\left(\eta \right)$.

**TABLE 8:** Required sample sizes for several specifications of the design priors $\pi^{D}\left(\theta \right)$ and $\pi^{D}\left(\eta \right)$, assuming the analysis priors $\pi^{A}\left(\theta \right)=\text{Beta}\left(1.8,0.2\right)$ and $\pi^{A}\left(\eta \right)=\text{Beta}\left(1.8,0.2\right)$.

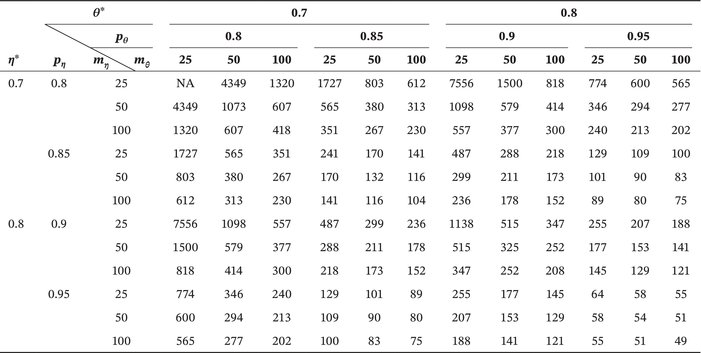

*Note:* $\theta^{*}$ and $\eta^{*}$ are the target values for sensitivity and specificity, respectively. $p_{\theta }$ and $p_{\eta }$ are the means of the design priors $\pi^{D}\left(\theta \right)$ and $\pi^{D}\left(\eta \right)$, respectively. $m_{\theta }$ and $m_{\eta }$ are the effective sample sizes of the design priors $\pi^{D}\left(\theta \right)$ and $\pi^{D}\left(\eta \right)$, respectively. The design prior $\pi^{D}\left(\rho \right)$ is fixed at $p_{\rho }=0.5$ and $m_{\rho }=100$. NA indicates that the target Bayesian power γ=0.8 is unattainable under the specified design priors $\pi^{D}\left(\theta \right)$ and $\pi^{D}\left(\eta \right)$.

## 6 Application Example

To illustrate how the proposed method can be applied in planning a future diagnostic accuracy study, we considered a hypothetical prospective study designed to independently validate the diagnostic performance of plasma PIVKA‐II for distinguishing patients with hepatocellular carcinoma (HCC) from healthy individuals, using the prespecified cutoff value of 35.60 mAU/mL. Feng et al. [16] reported the diagnostic performance of PIVKA‐II at this cutoff in 168 patients with HCC and 153 healthy subjects. Among the patients with HCC, 141 tested positive and 27 tested negative (sensitivity, 83.9%; 95% confidence interval, 77.6–88.7). Among the healthy subjects, 13 tested positive and 140 tested negative (specificity, 91.5%; 95% confidence interval, 86.0–95.0).

For this illustrative calculation, we assumed a future recruitment setting in which the proportion of disease‐positive participants was similar to the composition of the HCC and healthy‐control groups reported by Feng et al. Accordingly, we assumed the design prior $\pi^{D}\left(\rho \right)=\text{Beta}\left(168,153\right)$ (mode≈0.524). Based on the reported confidence interval, we assumed the design prior $\pi^{D}\left(\theta \right)=\text{Beta}\left(78.03,15.79\right)$ (mode≈0.839), such that the interval [0.75,0.9] corresponds to the 95% equal‐tail credible interval. Similarly, we assumed the design prior for specificity $\pi^{D}\left(\eta \right)=\text{Beta}\left(116.58,12.11\right)$ (mode≈0.912), such that the interval [0.85,0.95] corresponds to the 95% equal‐tail credible interval.

Table 9 shows the required sample sizes under varying analysis priors, when the target parameter values were $\theta^{*}=0.75$ and $\eta^{*}=0.8$, posterior probability thresholds were $\lambda_{\theta }=0.9$ and $\lambda_{\eta }=0.9$, and the Bayesian power threshold was γ=0.8. The target values $\theta^{*}$ and $\eta^{*}$ were treated as minimum acceptable performance values for the hypothetical validation study and were selected conservatively below the corresponding estimates and lower limits of the 95% confidence intervals reported by Feng et al. When both sensitivity and specificity were included in the success criterion, the required sample size ranged from 357 to 413 across the analysis‐prior specifications considered. In contrast, the values from 289 to 345 in the final column of Table 9 were based on sensitivity alone and therefore corresponded to a different study objective. For reference, when sample size determination was based on sensitivity alone and the sensitivity and prevalence were fixed at 0.839 and 0.524, respectively, the required sample sizes obtained using frequentist Approaches 1, 2, and 3 described in Section 4 were 181, 209, and 197, respectively.

**TABLE 9:** Required sample sizes for the PIVKA‐II application example for several specifications of the analysis priors $\pi^{A}\left(\theta \right)$ and $\pi^{A}\left(\eta \right)$.

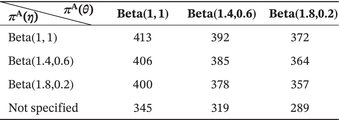

*Note:* “Not specified” indicates that no analysis prior $\pi^{A}\left(\eta \right)$ is assigned and that the success criterion is based on sensitivity alone.

## 7 Discussion

In this article, we proposed a Bayesian power‐based approach to sample size determination. The approach uses an analysis prior and a design prior for sensitivity and a design prior for prevalence. Bayesian power is defined as the prior predictive probability that the posterior probability of sensitivity exceeding the target value exceeds a prespecified threshold. We also showed that the method can be extended to specificity.

Under the numerical settings considered, the required sample size generally decreased as the design priors became more concentrated around their specified means. However, this pattern is not universal, because the effect of the effective sample size of the design prior depends on the location of its mean relative to the target value. When the design‐prior mean is above the target value, reducing the variance may increase the prior predictive probability of meeting the success criterion and thereby reduce the required sample size. Conversely, when the mean is close to or below the target value, reducing the variance may increase the required sample size or make the prespecified Bayesian power threshold unattainable. As shown in Section 4, under the parameter settings considered, the required sample size obtained using the proposed method was larger than that obtained using Approach 1 and smaller than those obtained using Approaches 2 and 3. Approach 1 used a normal approximation to the binomial distribution and yielded the smallest sample sizes in the settings examined. However, its apparently smaller sample size should be interpreted cautiously because the normal approximation may be inaccurate when the sample size is small or when the parameter value is close to the boundary of the parameter space. This is particularly relevant in diagnostic accuracy studies, where the anticipated and target values of sensitivity and specificity are often close to 1. Approach 2 avoids the normal approximation by using the exact binomial distribution to determine the required number of disease‐positive subjects, but obtains the total sample size by dividing this number by a fixed prevalence. In contrast, both Approach 3 and the proposed method account for the sampling variability in the observed number of disease‐positive subjects when evaluating their respective operating characteristics.

The smaller sample size obtained using the proposed method relative to Approach 3 has a specific mathematical explanation. As shown in Appendix B, under a degenerate design prior, the one‐sided exact binomial decision rule underlying Approach 3 is mathematically equivalent to the posterior‐probability criterion based on the formal limiting improper analysis prior $\pi^{A}\left(\theta \right)=\text{Beta}\left(0,1\right)$. Thus, Approach 3 can be recovered as a limiting special case of the proposed method. The proposed method in Table 5 instead uses the proper uniform analysis prior $\pi^{A}\left(\theta \right)=\text{Beta}\left(1,1\right)$, which yields a success region containing the rejection region of Approach 3. Consequently, under the conditions considered in Table 5, the Bayesian power is no smaller and the required sample size is no larger. This difference reflects the effect of the analysis prior and the corresponding decision rule and should not be interpreted as a general efficiency advantage of Bayesian methods over frequentist methods.

The comparison with frequentist sample size methods in Section 4 was intended as a reference comparison under fixed design assumptions. In that comparison, the degenerate design prior was used to align the Bayesian power‐based calculation with the fixed parameter values used in conventional frequentist power calculations. However, another possible approach is a mixed Bayesian/likelihood design, in which a non‐degenerate design prior is used at the design stage to represent uncertainty about the true diagnostic accuracy, whereas the final analysis is conducted using a frequentist criterion, such as a hypothesis test or confidence interval [17]. Such an approach can be useful when the final analysis is required to follow a frequentist framework, while the design stage aims to account for uncertainty in the assumed sensitivity, specificity, or prevalence. In the context of diagnostic accuracy studies, this would correspond to evaluating, under a design prior, the prior predictive probability that a future study will satisfy a frequentist success criterion. Although this is outside the primary scope of the present article, which focuses on a Bayesian posterior‐probability criterion at the analysis stage, it is a reasonable extension of the proposed method and may be useful in regulatory or confirmatory settings.

The unique feature of the analysis prior is that it can explicitly model the information obtained prior to the trial with respect to the unknown parameters. The analysis prior is updated based on data obtained during the trial, and inferences about unknown parameters are made based on the final posterior distribution to support decision making. This approach has several advantages over frequentist methods. In particular, it allows for the construction of success criteria for the trial based on posterior probabilities that are intuitively interpretable. In the frequentist approach, a clinically meaningful threshold is often used to specify a parameter value under the alternative hypothesis for power calculation. However, the rejection boundary of a statistical test is not necessarily identical to this clinically meaningful threshold. Consequently, a trial may be statistically significant even when the observed estimate does not reach the clinically meaningful threshold. Whether such outcomes are clinically meaningful is not always straightforward. In the proposed method, the design prior represents uncertainty about plausible values of diagnostic accuracy under the design scenario, and the success criteria of the trial are also explicitly defined based on posterior probabilities that are intuitively interpretable. This approach is expected to yield a trial design that is more persuasive from a clinical perspective. By contrast, the design prior can model uncertainty about the expected unknown parameter value. In the frequentist method, fixed values are assumed for the unknown parameters to enable power calculations. These values are usually determined based on results from external diagnostic trials, but there is no guarantee that they reflect the true parameter values. Therefore, the statistical power, a crucial value in sample size determination, may not be reliable. Misidentification of power carries the risk of failing the diagnostic trial due to insufficient sample size or causing ethical and financial problems due to excessive sample size. The design prior addresses this problem by incorporating uncertainty about the unknown parameter.

The proposed Bayesian power approach to sample size determination requires the following three priors: (1) analysis prior for sensitivity (or specificity), (2) design prior for sensitivity (or specificity), and (3) design prior for prevalence. As demonstrated in this study, when the effective sample size is constant, an optimistic analysis prior leads to a reduction in the required sample size, whereas a pessimistic analysis prior increases it. The analysis prior should be specified on the basis of any external information that is considered sufficiently relevant and reliable to contribute to the final posterior inference. It should not be selected mechanically to align with the target value $\theta^{*}$, a null value, or an anticipated alternative value. In the absence of such external information, we recommend the uniform Beta(1,1) distribution as a practical default analysis prior. Accordingly, because the historical information used to construct the design priors in the illustrative PIVKA‐II example was not considered sufficient to justify informative analysis priors for the final analysis of an independent validation study, we recommend a total sample size of 413, obtained using the uniform Beta(1,1) analysis priors for both sensitivity and specificity, when both measures are included in the success criterion. An informative analysis prior may be appropriate when relevant external evidence is available, but its construction and influence should be prespecified and justified, with discounting or robustification and sensitivity analyses considered where appropriate. When determining the design prior, it is important to base it on a “what‐if” spirit [8]. One approach is to first determine the most plausible value and then elicit expert judgments about the uncertainty surrounding that value (Section 3.1). Specifically, asking experts questions such as “What is the probability of exceeding (or falling below) a certain threshold?” can be an effective strategy. Next, eliciting information such as “The probability that the parameter is greater than l and less than u is α.” may be practical (Section 3.2). In practice, the elicited design prior should be presented graphically and reviewed with the experts to confirm that its location, dispersion, and tail probabilities adequately represent their intended judgments. Because experts may underestimate their uncertainty, the robustness of the resulting sample size should also be examined through sensitivity analyses over plausible design‐prior specifications [11]. Additionally, as an alternative to elicitation, it may also be feasible to directly incorporate the results of diagnostic trials conducted overseas into the design prior when introducing such trials into one's own country. A degenerate design prior may also be used to represent a fixed parameter assumption analogous to that used in conventional frequentist power calculations. In practice, it is usual to choose an objective and conservative scenario for the analysis prior and a slightly optimistic scenario for the design prior in order to gain stakeholder acceptance [8].

The asymptotic property described in Section 3.1 has an important practical implication for sample size determination. Wilson [18] noted that the assurance level used to determine the sample size should be chosen in light of the probability, under the design prior, that the parameter satisfies the success criterion, together with the context of the study. Accordingly, the feasibility of the prespecified Bayesian power threshold should be assessed under the chosen design prior before searching for the required sample size. If the threshold is not attainable, simply increasing the sample size will not resolve the problem, and the design assumptions or success criterion should be reconsidered.

It should be noted that the prevalence parameter does not relate to the performance of the diagnostic trial itself; it depends on the settings under which the diagnostic trial is performed. For example, when a diagnostic trial is performed on subjects visiting a hospital, the disease prevalence is expected to be relatively high. By contrast, when a large‐scale screening is performed, the disease prevalence is expected to be low. Therefore, when the sample size is determined using the proposed method, the design prior must be determined based on the setting in which the diagnostic trial will be conducted.

In this article, we focused only on a one‐sample design without interim monitoring. Interim monitoring based on posterior probability or posterior predictive probability could be incorporated into the Bayesian design [19]. If we wish to compare the performance measures of two diagnostic trials using data from two samples, sample size determination for unpaired data or paired data will be required. We intend to develop the two‐sample design for diagnostic accuracy comparison studies using the framework of the proposed method.

## Funding

The authors have nothing to report.

## Ethics Statement

The authors have nothing to report.

## Conflicts of Interest

The authors declare no conflicts of interest.

## Data Availability

Data sharing is not applicable to this article as no new data were created or analyzed in this study.

## Appendix A

The proposed method presented in this article has been made publicly available through an R Shiny application at https://bios‐kpum.shinyapps.io/BayesDAS/.

## Appendix B

We show that, in the setting of Table 5, the required sample size under the proposed method is no larger than that under Approach 3. Because both methods are evaluated under the same distribution of future data in this comparison, it suffices to compare their success regions for each possible $\left(n_{d},n_{s}\right)$. We set $\theta =\theta^{*}=\theta_{0}$.

Let $F_{n_{d}}\left(n_{s}|\theta \right)$ be the upper‐tail probability of a binomial distribution, and let $I_{\theta }\left(a,b\right)$ be the lower‐tail probability of a beta distribution. That is,

(A1)
$$F_{n_{d}}\left(n_{s}|\theta \right)=\sum_{x=n_{s}}^{n_{d}}f_{n_{d}}\left(x|\theta \right),\;I_{\theta }\left(a,b\right)=\int_{0}^{\theta }\frac{t^{a-1}{\left(1-t\right)}^{b-1}}{B\left(a,b\right)}\mathrm{dt}.$$

For $n_{s}\in \left\{1,2,\ldots ,n_{d}-1\right\}$, it follows that

(A2)
$$\begin{array}{cc} I_{\theta }\left(n_{s},1+n_{d}-n_{s}\right) & =\int_{0}^{\theta }\frac{t^{n_{s}-1}{\left(1-t\right)}^{n_{d}-n_{s}}}{B\left(n_{s},1+n_{d}-n_{s}\right)}\mathrm{dt}\\ & =\frac{n_{d}!}{\left(n_{s}-1\right)!\left(n_{d}-n_{s}\right)!}\left\{{\left[\frac{t^{n_{s}}{\left(1-t\right)}^{n_{d}-n_{s}}}{n_{s}}\right]}_{0}^{\theta }\right.\\ & \left.+\frac{n_{d}-n_{s}}{n_{s}}\int_{0}^{\theta }t^{n_{s}}{\left(1-t\right)}^{n_{d}-n_{s}-1}\mathrm{dt}\right\}\\ & =\frac{n_{d}!}{n_{s}!\left(n_{d}-n_{s}\right)!}\theta^{n_{s}}{\left(1-\theta \right)}^{n_{d}-n_{s}}\\ & +\int_{0}^{\theta }\frac{n_{d}!}{n_{s}!\left(n_{d}-n_{s}-1\right)!}t^{n_{s}}{\left(1-t\right)}^{n_{d}-n_{s}-1}\mathrm{dt}\\ & =f_{n_{d}}\left(n_{s}|\theta \right)+I_{\theta }\left(1+n_{s},n_{d}-n_{s}\right). \end{array}$$

Although $I_{\theta }\left(a,b\right)$ is not defined when one of its parameters becomes zero (as in the boundary cases $n_{s}=0$ or $n_{s}=n_{d}$ in Equation (A2)), the corresponding expressions can be consistently interpreted in the limiting sense, and the stated identity still holds for all $n_{s}\in \left\{0,1,\ldots ,n_{d}\right\}$. Applying the relation in Equation (A2), we obtain

(A3)
$$\begin{array}{cc} F_{n_{d}}\left(n_{s}|\theta \right) & =\sum_{x=n_{s}}^{n_{d}}f_{n_{d}}\left(x|\theta \right)\\ & =\sum_{x=n_{s}}^{n_{d}}\left\{I_{\theta }\left(x,1+n_{d}-x\right)-I_{\theta }\left(1+x,n_{d}-x\right)\right\}\\ & =I_{\theta }\left(n_{s},1+n_{d}-n_{s}\right). \end{array}$$

Let $c^{F}\left(n_{d}\right)$ be the critical value under Approach 3 with fixed $n_{d}$. That is, from Equation (A3),

(A4)
$$\begin{array}{cc} c^{F}\left(n_{d}\right) & =\min\left\{n_{s}\in \mathbb{N}|F_{n_{d}}\left(n_{s}|\theta \right)\leq 1-\lambda_{\theta }\right\}\\ & =\min\left\{n_{s}\in \mathbb{N}|I_{\theta }\left(n_{s},1+n_{d}-n_{s}\right)\leq 1-\lambda_{\theta }\right\}. \end{array}$$

Let $c^{B\left(0,1\right)}\left(n_{d}\right)$ be the critical value under the proposed method with fixed $n_{d}$, using the analysis prior $\pi^{A}\left(\theta \right)=\text{Beta}\left(\varepsilon ,1\right)$, and take the formal limit as ε↓0. This limiting prior is conventionally denoted by the improper prior $\pi^{A}\left(\theta \right)=\text{Beta}\left(0,1\right)$. For $n_{s}>1$, the limiting posterior distribution is $\theta \;|\;n_{d},n_{s}∼\text{Beta}\left(n_{s},1+n_{d}-n_{s}\right)$. Therefore,

(A5)
$$\begin{array}{cc} c^{B\left(0,1\right)}\left(n_{d}\right) & =\min\left\{n_{s}\in \mathbb{N}|\mathrm{Pr}\left(\theta >\theta^{*}|n_{d},n_{s}\right)>\lambda_{\theta }\right\}\\ & =\min\left\{n_{s}\in \mathbb{N}|I_{\theta }\left(n_{s},1+n_{d}-n_{s}\right)\leq 1-\lambda_{\theta }\right\}. \end{array}$$

From Equations (A4) and (A5), it follows that, for every $n_{d}$, the limiting Bayesian success region coincides with the rejection region of the one‐sided exact binomial test. Although Beta(0,1) is improper, it is used here only as a formal limiting prior to establish the relationship between the two decision rules. When $n_{s}=0$, the posterior under Beta(ε,1) concentrates at zero as ε↓0, so the limiting posterior probability of exceeding any $\theta^{*}$ is zero. Hence, $n_{s}=0$ does not belong to the success region.

Next, let $c^{B\left(1,1\right)}\left(n_{d}\right)$ be the critical value under the proposed method with fixed $n_{d}$, using the analysis prior $\pi^{A}\left(\theta \right)=\text{Beta}\left(1,1\right)$. As described in Section 2.2,

(A6)
$$\begin{array}{cc} c^{B\left(1,1\right)}\left(n_{d}\right) & =\min\left\{n_{s}\in \mathbb{N}|\mathrm{Pr}\left(\theta >\theta^{*}|n_{d},n_{s}\right)>\lambda_{\theta }\right\}\\ & =\min\left\{n_{s}\in \mathbb{N}|I_{\theta }\left(1+n_{s},1+n_{d}-n_{s}\right)\leq 1-\lambda_{\theta }\right\}. \end{array}$$

For every possible $\left(n_{d},n_{s}\right)$, the $\text{Beta}\left(1+n_{s},1+n_{d}-n_{s}\right)$ distribution is stochastically larger than the limiting $\text{Beta}\left(n_{s},1+n_{d}-n_{s}\right)$ distribution. Thus,

(A7)
$$I_{\theta }\left(n_{s},1+n_{d}-n_{s}\right)>I_{\theta }\left(1+n_{s},1+n_{d}-n_{s}\right),$$

Taking the minimum over $n_{s}\in \mathbb{N}$ then gives

(A8)
$$\begin{array}{cc} & \min\left\{n_{s}\in \mathbb{N}|I_{\theta }\left(1+n_{s},1+n_{d}-n_{s}\right)\leq 1-\lambda_{\theta }\right\}\\ & \;\leq \min\left\{n_{s}\in \mathbb{N}|I_{\theta }\left(n_{s},1+n_{d}-n_{s}\right)\leq 1-\lambda_{\theta }\right\}, \end{array}$$

and therefore:

(A9)
$$c^{B\left(1,1\right)}\left(n_{d}\right)\leq c^{B\left(0,1\right)}\left(n_{d}\right)=c^{F}\left(n_{d}\right).$$

Since Equation (A9) holds for all $n_{d}\in \left\{0,1,\ldots ,n\right\}$, the success criterion of the proposed method is always equivalent to, or less stringent than, that of Approach 3. Consequently, under the parameter settings considered in Table 5, the Bayesian power of the proposed method is no smaller than that of Approach 3, and the corresponding required sample size is no larger.
